# Supplementary material for: A deep learning-based toolbox for Automated Limb Motion Analysis (ALMA) in murine models of neurological disorders
Source: Commun Biol. 2022 Feb 15;5:131. doi: 10.1038/s42003-022-03077-6 (PMC8847458; doi:10.1038/s42003-022-03077-6)
Supplement: Supplementary file 2 — Supplementary Information [file 42003_2022_3077_MOESM2_ESM.pdf]

Supplementary Information

A deep learning-based toolbox for Automated Limb Motion Analysis (ALMA) in murine models of neurological disorders

Almir Aljovic[1,2,3,#], Shuqing Zhao[1,2,3,#], Maryam Chahin[1,2,3], Clara de la Rosa del Val[1,2,3], Valerie VanSteenbergen[1,2], Martin Kerschensteiner[1,2,4], and Florence M Bareyre[1,2,4]

# Contributed equally

1 Institute of Clinical Neuroimmunology, University Hospital, LMU Munich, 81377 Munich, Germany
2 Biomedical Center Munich (BMC), Faculty of Medicine, LMU Munich, 82152 Planegg-Martinsried, Germany
3 Graduate School of Systemic Neurosciences, Ludwig-Maximilians-Universitaet Munich, 82152 Planegg-Martinsried, Germany
4 Munich Cluster of Systems Neurology (SyNergy), 81377 Munich, Germany

Correspondence should be addressed to:
Florence M. Bareyre: florence.bareyre@med.uni-muenchen.de

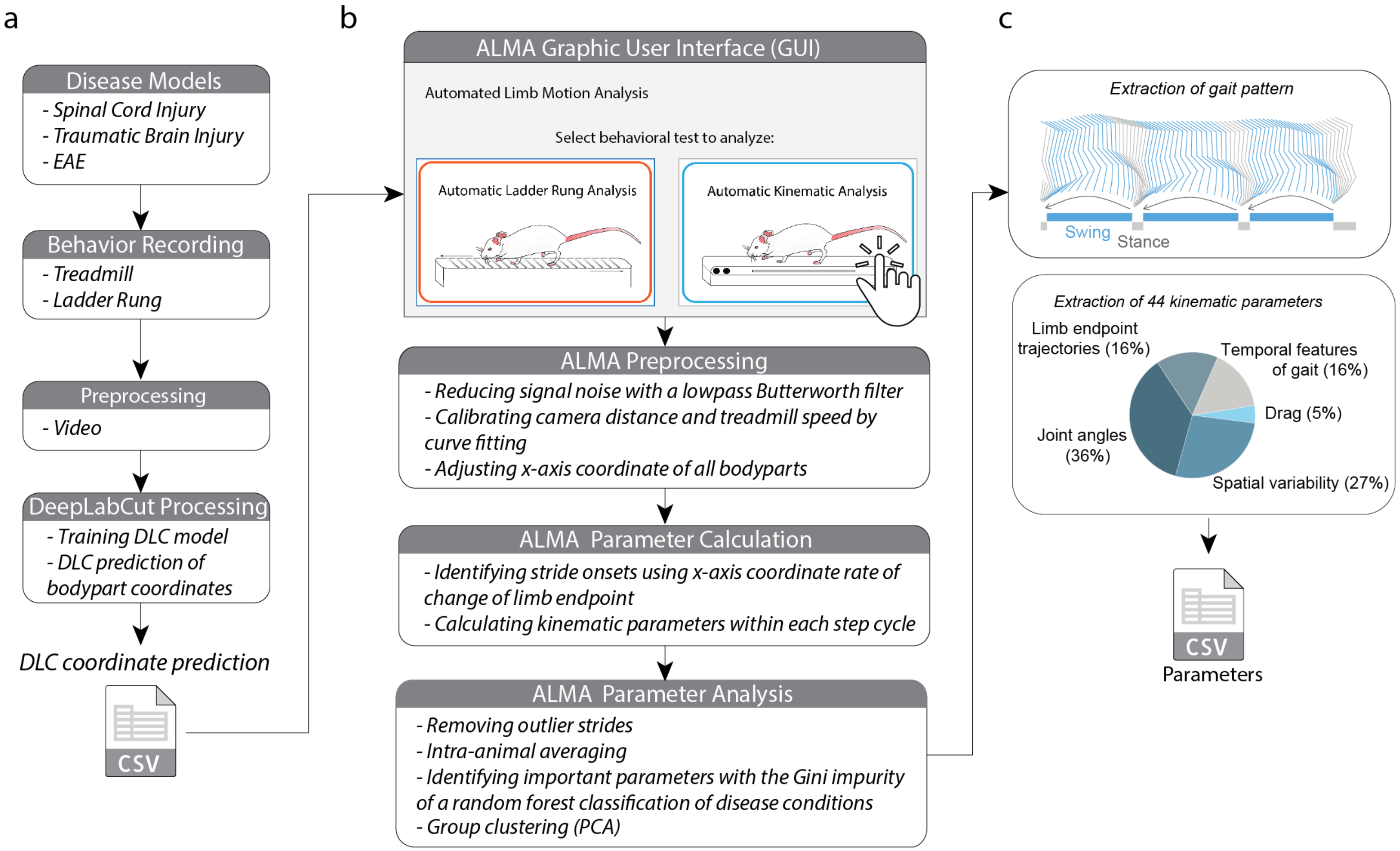

Supplementary Figure 1: Detailed flowchart of the ALMA computations used to obtain kinematic parameters

a- Flowchart of the experiment, including selection of injury paradigm, behavior recording, video preprocessing, DeepLabCut (DLC) markerless labeling, model training, and coordinate export. **b-** Workflow of the ALMA toolbox, with a user-friendly graphical user interface (GUI) for choosing kinematic analysis, and the computational steps used to identify important parameters and perform group clustering. **c-** ALMA allows the user to extract gait patterns and analyze the 44 parameters.

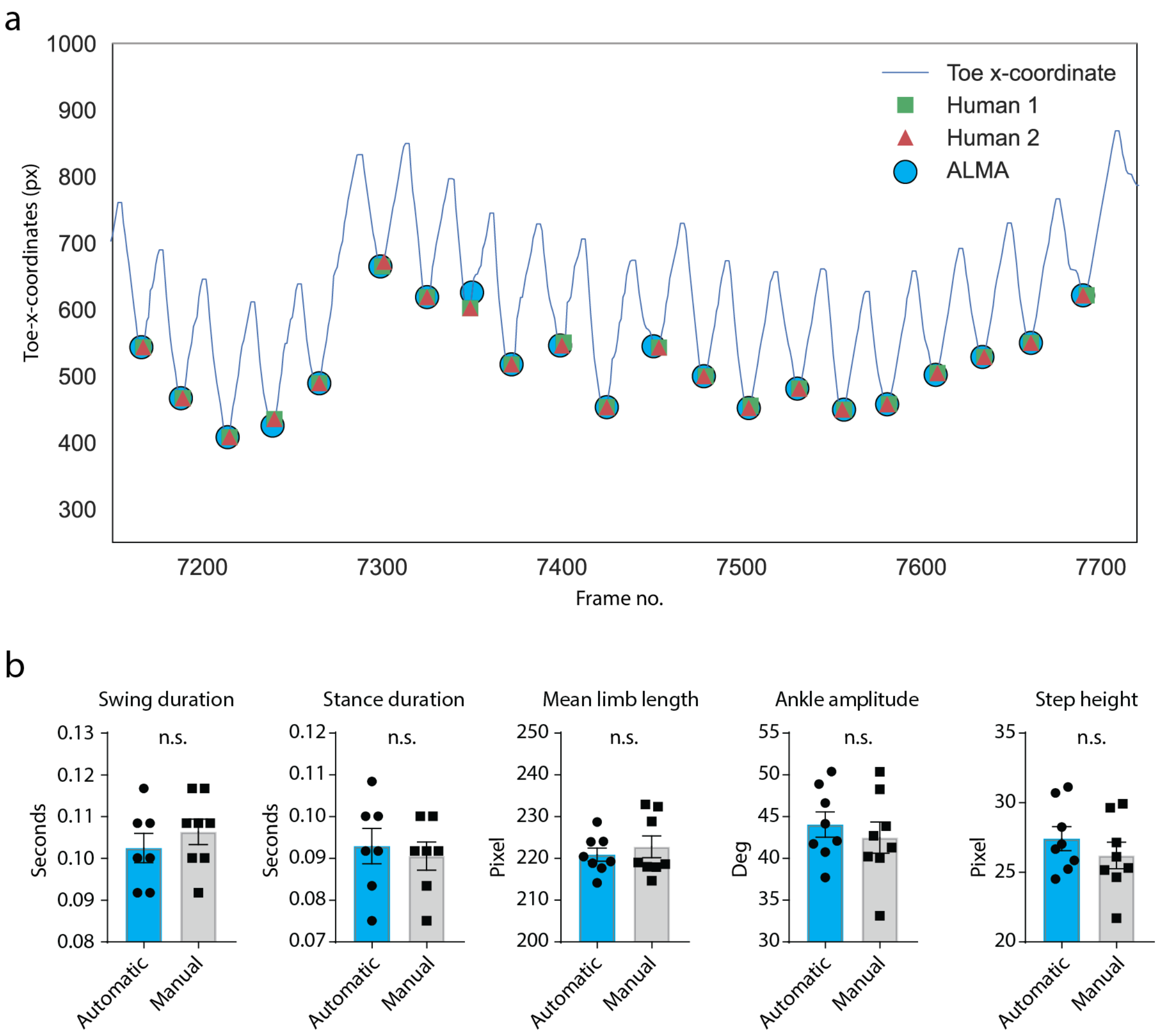

Supplementary Figure 2: Validation of gait parameters computed with ALMA

a- Comparison of the step cycle onset determination using either the ALMA toolbox (black dots) or two independent investigator performing the detection manually and separately (green square and red triangle). **b-** Comparison of the automatic calculation of 5 gait parameters using ALMA and of the manual validation (blue: automated detection with ALMA; grey: manual analysis).

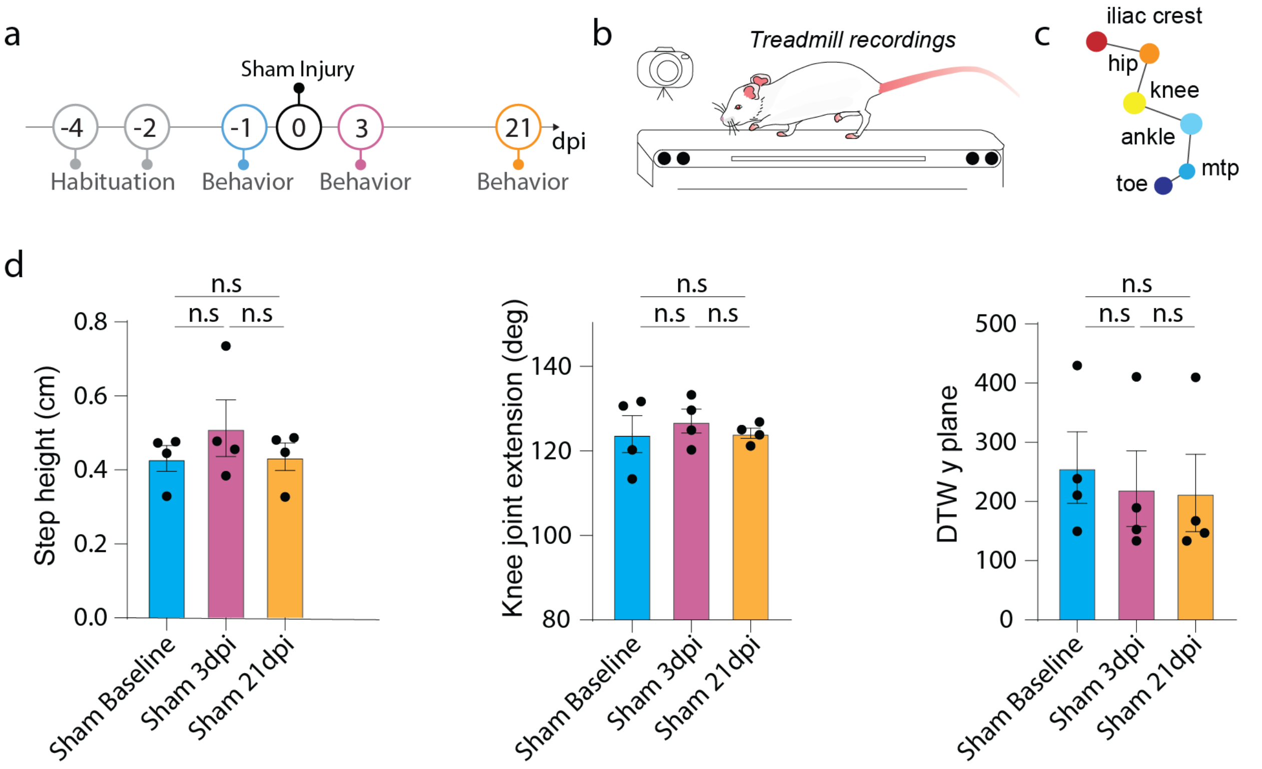

Supplementary Figure 3: ALMA analysis does not detect any changes of gait in sham injured mice tested on the treadmill

a- Timeline of the sham experiment. **b-** Schematic of the treadmill system used to record the behavior of mice. **c-** Schematic of DeepLabCut (DLC) markerless joint labeling. Six joints were labeled: iliac crest, hip, knee, ankle, metatarsophalangeal joint (MTP), and toe. **d-** Quantitative evaluation of the same parameters as in Figure 2, such as step height, knee joint extension, or dynamic time warping (DTW), at baseline, 3 and 21 days post-sham surgery. Repeated one-way ANOVA was used to analyze knee joint extension ($p = 0.440$; $n = 4$), step height ($p=0.458$; $n=4$) and DTW y plane ($p=850$; $n = 4$). In all panels, data are presented as mean± SEM; *$p < 0.05$; **$p < 0.01$; ***$p < 0.001$. dpi: days post-injury.

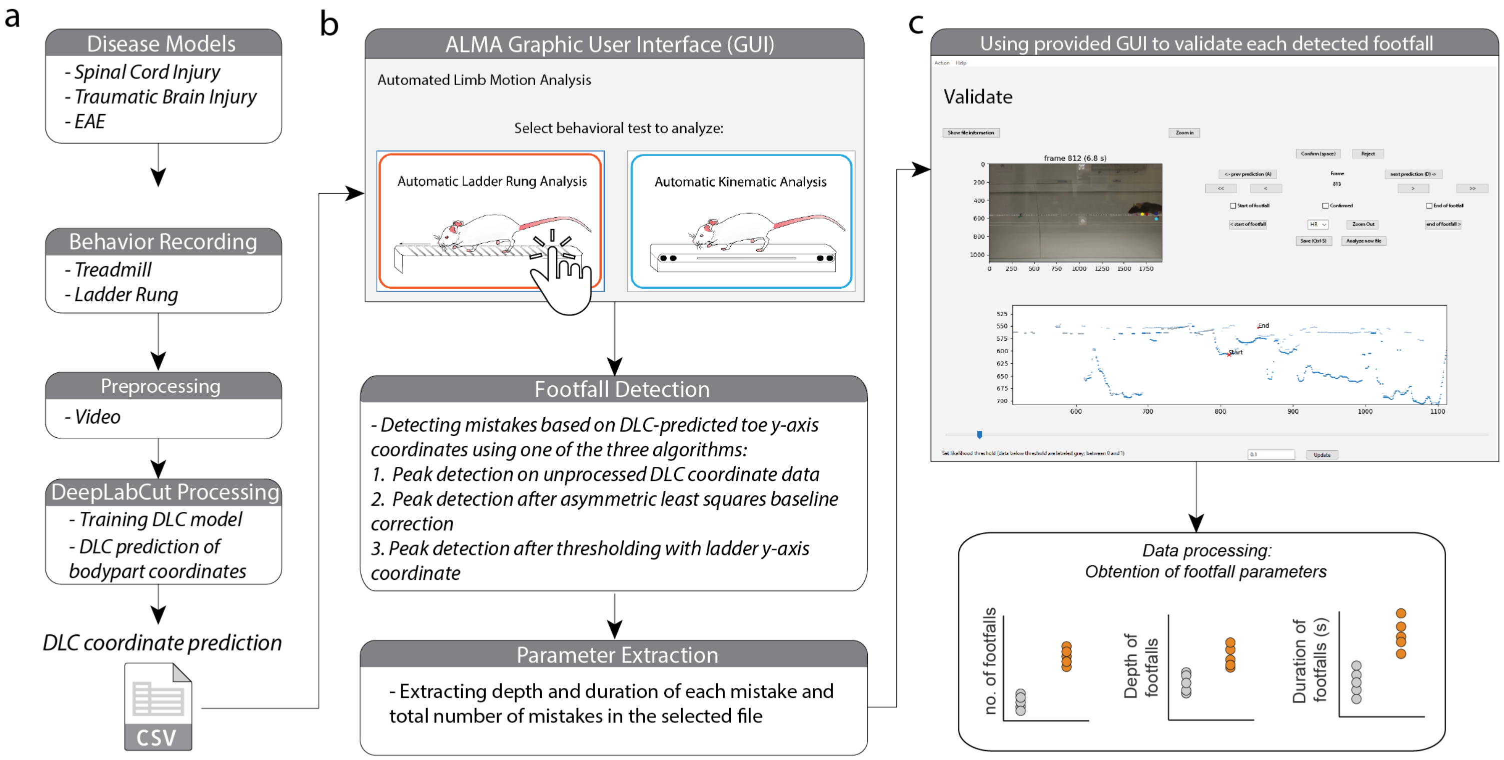

Supplementary Figure 4: Detailed flowchart of ALMA computations used to obtain footfall parameters

a- Flowchart of the experiment, including choice of injury paradigm, behavioral recording, video preprocessing, DeepLabCut (DLC) markerless labeling, model training, and coordinate export. **b-** Workflow of the ALMA toolbox, with a user-friendly graphical user interface (GUI) for selecting ladder rung analysis, and computational steps to identify the number, depth, and duration of footfalls. **c-** Each detected footfall can be visualized and manually validated or excluded before final data are generated by ALMA.

Parameter clusters	Parameters	Parameter Calculation
Temporal features of gait	stance duration (s)	(Frame no. at start of swing phase – frame no. at start of step cycle) / frame rate
	swing duration (s)	(Frame no. at end of step cycle – frame no. at end of stance phase) / frame rate
	swing percentage (%)	no. frames of swing phase / step cycle duration
	stance percentage (%)	no. frames of stance phase / step cycle duration
	max velocity during swing (cm/s)	maximum endpoint velocity during step cycle
	cycle duration (s)	(step cycle end frame no. – step cycle start frame no.) / Frame Rate
	cycle duration (# frames)	correction
Limb endpoint trajectories	cycle velocity (cm/s)	stride length (cm) / [cycle duration (no. frames) * frame rate (frames / s)]
	stride length (cm)	((toe x coordinate at step cycle end) – (toe x coordinate at step cycle start)) / pixel-to-centimeter ratio
	mean toe-to-crest distance (cm)	mean of 2D Euclidean distance from toe to iliac crest during the step cycle
	max toe-to-crest distance (cm)	Maximum 2D Euclidean distance from toe to iliac crest during the step cycle
	min toe-to-crest distance (cm)	Minimum 2D Euclidean distance from toe to iliac crest during the step cycle
	toe-to-crest distance SD (cm)	Standard deviation of 2D Euclidean distance from toe to iliac crest during the step cycle
	step height (cm)	(treadmill y coordinate – minimum toe y coordinate during step cycle) / px-to-cm ratio
Drag	drag duration (s)	(no. frames where Butterworth filtered toe y coordinate > treadmill y coordinate) / frame rate
	drag percentage (%)	drag duration / swing duration
Joint angles	mtp joint extension (deg)	maximum MTP joint angle within step cycle
	mtp joint flexion (deg)	minimum MTP joint angle within step cycle
	mtp joint amplitude (deg)	Maximum – minimum MTP joint angle within step cycle
	mtp joint SD (deg)	Standard deviation of MTP joint angles within step cycle
	ankle joint extension (deg)	maximum ankle joint angle within step cycle
	ankle joint flexion (deg)	minimum ankle joint angle within step cycle
	ankle joint amplitude (deg)	Maximum – minimum ankle joint angle within step cycle
	ankle joint SD (deg)	Standard deviation of ankle joint angles within step cycle
	knee joint extension (deg)	maximum knee joint angle within step cycle
	knee joint flexion (deg)	minimum knee joint angle within step cycle
	knee joint amplitude (deg)	Maximum – minimum knee joint angle within step cycle
	knee joint SD (deg)	Standard deviation of knee joint angles within step cycle
	hip joint extension (deg)	maximum hip joint angle within step cycle
	hip joint flexion (deg)	minimum hip joint angle within step cycle
	hip joint amplitude (deg)	Maximum – minimum hip joint angle within step cycle
	hip joint SD (deg)	Standard deviation of hip joint angles within step cycle
Spatial variability	DTW distance x plane 5 strides mean	mean of x coordinate (1D) DTW distance of 5 adjacent valid strides
	DTW distance x plane 5 strides SD	standard deviation of x coordinate (1D) DTW distance of 5 adjacent valid strides
	DTW distance y plane 5 strides mean	mean of y coordinate (1D) DTW distance of 5 adjacent valid strides
	DTW distance y plane 5 strides SD	standard deviation of y coordinate (1D) DTW distance of 5 adjacent valid strides
	DTW distance xy plane 5 strides mean	mean of x-y coordinate (2D) DTW distance of 5 adjacent valid strides
	DTW distance xy plane 5 strides SD	standard deviation of x-y coordinate (2D) DTW distance of 5 adjacent valid strides
	DTW distance x plane 10 strides mean	mean of x coordinate (1D) DTW distance of 10 adjacent valid strides
	DTW distance x plane 10 strides SD	standard deviation of x coordinate (1D) DTW distance of 10 adjacent valid strides
	DTW distance y plane 10 strides mean	mean of y coordinate (1D) DTW distance of 10 adjacent valid strides
	DTW distance y plane 10 strides SD	standard deviation of y coordinate (1D) DTW distance of 10 adjacent valid strides
	DTW distance xy plane 10 strides mea	mean of x-y coordinate (2D) DTW distance of 10 adjacent valid strides
	DTW distance xy plane 10 strides SD	standard deviation of x-y coordinate (2D) DTW distance of 10 adjacent valid strides

Supplementary Table 1: Description and mathematical formulae for parameters calculated by the ALMA toolbox.

Parameter clusters	Parameters	Baseline		1DPI		10DPI	
		Mean	SD	Mean	SD	Mean	SD
Temporal features of gait	stance duration (s)	0.272501	0.02778	0.324468	0.073975	0.278786	0.070118
	swing duration (s)	32.66743	3.330316	38.89727	8.868065	33.42085	8.405765
	swing percentage (%)	20.01785	2.378841	13.24887	6.097586	17.69423	5.598283
	stance percentage (%)	5.030653	0.438425	3.736301	1.115117	4.370403	0.794919
	max velocity during swing (cm/s)	0.128701	0.022238	0.154144	0.038008	0.131397	0.029552
	cycle duration (s)	0.1438	0.01839	0.170325	0.04709	0.147389	0.04209
	cycle duration (no. frames)	0.531433	0.05752	0.521877	0.040124	0.519481	0.034092
Limb endpoint trajectories	cycle velocity (cm/s)	0.468567	0.05752	0.478123	0.040124	0.480519	0.034092
	stride length (cm)	2.479889	0.449962	2.395532	0.286082	2.627573	0.375719
	mean toe-to-crest distance (cm)	3.053605	0.345247	2.819122	0.346363	3.044387	0.365592
	max toe-to-crest distance (cm)	2.006263	0.492077	2.083746	0.247717	2.274772	0.372092
	min toe-to-crest distance (cm)	0.316346	0.065982	0.226115	0.059471	0.239906	0.094296
	toe-to-crest distance SD (cm)	0.418506	0.082368	0.129611	0.071069	0.357871	0.253824
	step height (cm)	53.82473	4.310894	41.64993	16.02726	48.72549	9.950884
Drag	drag duration (s)	172.4974	4.808273	165.2026	9.880856	169.1852	4.10966
	drag percentage (%)	110.9118	25.12176	111.1831	8.887672	120.9324	13.46444
Joint angles	mtp joint extension (deg)	61.58558	21.94498	54.01952	8.439814	48.25288	13.17755
	mtp joint flexion (deg)	21.04034	9.049714	17.2016	2.713314	15.7355	4.239146
	mtp joint amplitude (deg)	115.2692	5.197123	127.5739	19.01481	115.0247	10.9949
	mtp joint SD (deg)	66.17388	14.55343	72.41404	20.717	68.1328	12.41157
	ankle joint extension (deg)	49.09534	16.15845	55.15981	12.69716	46.89194	9.688858
	ankle joint flexion (deg)	16.1417	4.982632	18.40307	4.211443	15.79921	3.228546
	ankle joint amplitude (deg)	126.2849	6.819535	103.8706	13.36608	126.2695	6.818621
	ankle joint SD (deg)	84.43803	15.08882	76.30352	13.38735	95.49433	8.506513
	knee joint extension (deg)	41.84686	14.68188	27.56712	4.866101	30.77513	11.23552
	knee joint flexion (deg)	14.05208	4.855436	8.731922	1.816848	9.909117	3.596147
	knee joint amplitude (deg)	101.0559	15.50737	98.99893	10.52836	108.8717	16.53888
	knee joint SD (deg)	59.93486	6.361746	65.05851	11.32375	73.21607	6.771436
	hip joint extension (deg)	41.12103	15.67308	33.94042	10.02455	35.65567	12.97713
	hip joint flexion (deg)	14.0558	5.617646	11.42244	3.801759	12.06421	4.75993
	hip joint amplitude (deg)	0.002212	0.001304	0.008046	0.008611	0.003418	0.003746
	hip joint SD (deg)	0.004198	0.002956	0.044267	0.049751	0.014499	0.019797
Spatial variability	DTW distance x plane 5 strides mean	846.4958	459.622	881.9535	240.2091	825.1326	359.3023
	DTW distance x plane 5 strides SD	672.7041	528.8476	672.214	280.0555	621.6206	364.4608
	DTW distance y plane 5 strides mean	390.5996	356.6238	187.8594	208.1919	336.4633	414.1873
	DTW distance y plane 5 strides SD	366.6243	419.538	165.2883	231.8537	281.3317	412.1286
	DTW distance xy plane 5 strides mean	1114.693	641.9847	1011.349	295.3583	1064.549	581.26
	DTW distance xy plane 5 strides SD	842.2055	728.3903	745.0359	348.713	744.0106	551.4335
	DTW distance x plane 10 strides mean	850.8711	467.0968	847.5654	241.5939	852.2332	403.8618
	DTW distance x plane 10 strides SD	932.5275	836.09	836.4553	423.1833	862.8633	607.68
	DTW distance y plane 10 strides mean	393.4395	361.2572	189.993	211.1147	345.7809	439.7726
	DTW distance y plane 10 strides SD	540.5131	687.5086	243.453	377.3961	414.5704	667.7815
	DTW distance xy plane 10 strides mear	1120.609	654.5719	979.3329	302.8977	1097.534	629.7265
	DTW distance xy plane 10 strides SD	1180.169	1176.488	950.7398	559.1539	1044.252	898.6512

Supplementary Table 2: Quantitative measurement of 44 spinal cord injury kinematic parameters obtained by ALMA at baseline, 3 dpi, and 21 dpi. Data are presented as mean ± SD.

Parameter clusters	Parameters	Baseline		1DPI		10DPI	
		Mean	SD	Mean	SD	Mean	SD
Temporal features of gait	stance duration (s)	0.121824	0.015986	0.118299	0.013777	0.120694	0.010487
	swing duration (s)	0.114017	0.009108	0.132097	0.019376	0.119472	0.013917
	swing percentage (%)	0.488084	0.036879	0.520157	0.040942	0.494563	0.028555
	stance percentage (%)	0.511916	0.036879	0.479843	0.040942	0.505437	0.028555
	max velocity during swing (cm/s)	60.76525	4.108749	56.56078	4.905492	59.04529	5.090353
	cycle duration (s)	0.235841	0.018705	0.250396	0.020603	0.240166	0.021154
	cycle duration (no. frames)	28.27267	2.242367	30.0175	2.469888	28.79111	2.535994
Limb endpoint trajectories	cycle velocity (cm/s)	24.03619	1.860084	21.56867	2.675134	23.06046	2.2548
	stride length (cm)	5.467224	0.185781	5.049387	0.356628	5.303987	0.263472
	mean toe-to-crest distance (cm)	2.697055	0.07725	2.629069	0.187952	2.749006	0.154402
	max toe-to-crest distance (cm)	3.040595	0.085559	3.046766	0.208821	3.128813	0.175387
	min toe-to-crest distance (cm)	2.308645	0.08491	2.265401	0.166957	2.341604	0.170356
	toe-to-crest distance SD (cm)	0.226423	0.024947	0.233403	0.029264	0.243264	0.027067
	step height (cm)	0.470995	0.033842	0.364227	0.064624	0.454162	0.045857
Drag	drag duration (s)	0.002077	0.003029	0.005172	0.004947	0.002338	0.001843
	drag percentage (%)	0.003694	0.005337	0.012383	0.00921	0.004401	0.003797
Joint angles	mtp joint extension (deg)	175.5036	1.753417	174.4127	2.896869	173.6816	4.232018
	mtp joint flexion (deg)	123.8531	5.63525	121.8831	8.1265	119.2426	2.745881
	mtp joint amplitude (deg)	51.65052	5.020858	52.52962	6.924255	54.43905	5.164657
	mtp joint SD (deg)	17.18208	1.634765	17.42373	2.517821	18.12524	1.821725
	ankle joint extension (deg)	113.137	3.532662	110.5983	4.017837	116.4725	3.75105
	ankle joint flexion (deg)	72.58927	6.516049	70.93998	4.489512	71.86161	3.429244
	ankle joint amplitude (deg)	40.54775	6.41054	39.65832	5.788168	44.61088	1.698437
	ankle joint SD (deg)	13.77948	2.039465	13.10895	2.07707	15.14024	0.585989
	knee joint extension (deg)	126.6993	3.974741	125.9935	4.241151	125.8478	6.535263
	knee joint flexion (deg)	97.21903	6.4127	93.84855	2.810883	93.93848	5.141963
	knee joint amplitude (deg)	29.48023	3.394054	32.14492	5.872531	31.90928	3.259213
	knee joint SD (deg)	10.19764	1.280341	10.94247	1.958866	10.78471	1.174599
	hip joint extension (deg)	103.7711	5.299289	102.7507	5.37069	103.9755	5.317694
	hip joint flexion (deg)	66.78887	4.757523	68.67242	2.336178	68.20187	4.316921
	hip joint amplitude (deg)	36.98223	4.181746	34.07829	5.372466	35.77363	5.799337
	hip joint SD (deg)	12.54367	1.501702	11.5638	2.17545	12.27671	2.055408
Spatial variability	DTW distance x plane 5 strides mean	540.3867	212.6678	917.2769	398.675	534.6435	104.9336
	DTW distance x plane 5 strides SD	327.3784	158.1207	733.6116	448.5399	324.158	116.3877
	DTW distance y plane 5 strides mean	166.5777	69.33384	437.2623	299.5536	180.2077	101.5493
	DTW distance y plane 5 strides SD	121.3037	67.77559	431.9553	348.5609	119.0592	81.1103
	DTW distance xy plane 5 strides mean	678.5163	252.6987	1203.494	534.4544	700.3912	178.0202
	DTW distance xy plane 5 strides SD	373.6489	178.48	941.5367	599.772	378.0759	135.8761
	DTW distance x plane 10 strides mean	506.2561	178.4697	864.9111	331.0459	507.8059	99.62951
	DTW distance x plane 10 strides SD	373.0266	190.7426	937.0467	579.7646	386.655	154.4982
	DTW distance y plane 10 strides mean	160.743	69.26296	421.6389	268.3219	179.0769	98.20865
	DTW distance y plane 10 strides SD	156.472	104.2623	618.8024	497.2211	144.4497	102.226
	DTW distance xy plane 10 strides mean	639.7849	222.8888	1146.656	459.0672	674.2344	179.8192
	DTW distance xy plane 10 strides SD	435.1609	228.9883	1244.121	801.8841	455.6019	177.9623

Supplementary Table 3: Quantitative measurement of 44 traumatic brain injury kinematic parameters obtained by ALMA at baseline, 1 dpi, and 10 dpi. Data are presented as mean ± SD.

Parameter clusters	Parameters	Baseline		Onset		Peak		Recovery	
		Mean	SD	Mean	SD	Mean	SD	Mean	SD
Temporal features of gait	stance duration (s)	0.146172	0.021181	0.166544	0.034631	0.041576	0.085221	0.117007	0.094296
	swing duration (s)	0.143492	0.02733	0.22568	0.101962	0.088798	0.179719	0.165939	0.144439
	swing percentage (%)	0.495995	0.028928	0.529939	0.056731	0.145752	0.289219	0.37551	0.299778
	stance percentage (%)	0.504005	0.028928	0.470061	0.056731	0.07647	0.151742	0.291157	0.236909
	max velocity during swing (cm/s)	55.23709	6.070852	43.76049	14.91701	5.247924	11.64308	17.98196	16.41625
	cycle duration (s)	0.289664	0.04528	0.392224	0.125119	0.130375	0.264851	0.282946	0.227153
	cycle duration (no. frames)	34.72497	5.428173	47.01979	14.99922	15.62934	31.75039	33.9196	27.23115
Limb endpoint trajectories	cycle velocity (cm/s)	21.32164	3.180192	13.95752	6.834312	1.151734	2.285712	4.397505	3.680455
	stride length (cm)	5.489372	0.458125	4.2186	1.133514	0.633855	1.285671	1.615061	1.358392
	mean toe-to-crest distance (cm)	2.898692	0.104501	2.625188	0.391537	4.351212	1.279366	2.550672	0.885827
	max toe-to-crest distance (cm)	3.380931	0.148423	3.167334	0.378405	0.810533	1.608925	1.794892	1.457816
	min toe-to-crest distance (cm)	2.405055	0.099778	2.155158	0.37814	0.577336	1.15541	1.134799	0.89133
	toe-to-crest distance SD (cm)	0.295517	0.042003	0.300388	0.06008	4.123457	2.43996	1.095595	1.277788
	step height (cm)	0.507989	0.088267	0.459763	0.316867	0.029749	0.074518	0.11031	0.109696
Drag	drag duration (s)	0.001847	0.001666	0.009414	0.008313	0.792686	0.411622	0.35366	0.501278
	drag percentage (%)	0.003411	0.003624	0.024853	0.026162	0.812266	0.372855	0.388467	0.475522
Joint angles	mtp joint extension (deg)	172.4644	5.188135	174.9312	2.198022	37.61502	74.64835	116.9898	90.67581
	mtp joint flexion (deg)	114.7451	4.99702	118.5715	8.143236	25.38119	50.97598	78.39111	68.54693
	mtp joint amplitude (deg)	57.7193	2.620203	56.35964	8.379902	12.23383	25.88711	38.59871	43.29083
	mtp joint SD (deg)	18.76039	0.564562	17.79579	2.016047	3.556986	7.498147	12.01286	13.35917
	ankle joint extension (deg)	125.5605	4.080133	121.0429	9.008408	34.3132	68.67215	75.6245	64.88876
	ankle joint flexion (deg)	71.97364	3.663684	63.82674	14.01654	16.00861	36.60254	32.20136	27.09573
	ankle joint amplitude (deg)	53.58688	3.543148	57.21616	12.92742	18.30459	37.48068	43.42315	41.96718
	ankle joint SD (deg)	17.66814	0.666776	18.39213	3.990212	5.757634	12.07673	14.16803	13.79047
	knee joint extension (deg)	129.3757	8.336228	123.4652	12.36152	30.03759	59.64383	60.33684	67.80817
	knee joint flexion (deg)	98.84365	8.789085	86.01011	11.70408	13.77771	27.39063	38.80837	44.38005
	knee joint amplitude (deg)	30.53206	2.168625	37.45506	11.33907	16.25987	32.26862	26.04695	21.51931
	knee joint SD (deg)	9.745117	0.811088	11.41107	2.957107	4.586983	9.123891	7.766365	6.494424
	hip joint extension (deg)	108.181	9.849253	111.5816	17.06113	28.04826	56.10665	79.83668	63.1816
	hip joint flexion (deg)	68.45421	7.654095	69.0225	12.13957	12.57397	24.9621	57.7005	46.57007
	hip joint amplitude (deg)	39.7268	6.568989	42.55913	8.897696	15.47429	31.69255	22.13618	18.49006
	hip joint SD (deg)	13.30313	2.258298	13.76168	2.435751	4.489437	9.219224	7.271831	6.142711
Spatial variability	DTW distance x plane 5 strides mean	45857.23	11021.48	36800.65	9766.16	7474.34	15456.18	21758.33	21287.55
	DTW distance x plane 5 strides SD	32826.21	10174.48	25932.21	8784.094	4711.648	9523.548	16527.97	17595.52
	DTW distance y plane 5 strides mean	1101.058	864.0492	1315.66	1528.281	297.5328	614.0889	1178.543	1781.837
	DTW distance y plane 5 strides SD	1138.067	1038.172	1441.36	1879.303	295.5045	606.3933	1329.691	2207.134
	DTW distance xy plane 5 strides mean	46079.74	11160.51	37059.56	9888.306	7530.001	15579.8	21903.38	21442.29
	DTW distance xy plane 5 strides SD	32831.51	10118.97	26047.36	8881.842	4737.676	9574.174	16642.99	17773.13
	DTW distance x plane 10 strides mean	83589.18	20562.34	67016.07	17302.3	13693.9	28453.32	35830.49	34471.14
	DTW distance x plane 10 strides SD	66035.47	20131.86	54801.45	18180.48	10072.3	20333.21	28886.19	27767.73
	DTW distance y plane 10 strides mean	1227.397	1005.883	1597.406	2151.07	410.9481	832.2065	671.244	902.5316
	DTW distance y plane 10 strides SD	1655.829	1508.576	2385.219	3425.706	536.9708	1069.159	1002.274	1499.278
	DTW distance xy plane 10 strides mean	83770.21	20630.15	67359.04	17446.34	13746.2	28563.51	35895.63	34523.01
	DTW distance xy plane 10 strides SD	65996.02	20080.61	54917.09	18265.47	10117.34	20421.81	28961.03	27854.55

Supplementary Table 4: Quantitative measurement of 44 EAE kinematic parameters obtained by ALMA at baseline, onset, peak, and disease recovery. Data are presented as mean ± SD.
