# Supplementary material for: A deep learning-based toolbox for Automated Limb Motion Analysis (ALMA) in murine models of neurological disorders
Source: Commun Biol. 2022 Feb 15;5:131. doi: 10.1038/s42003-022-03077-6 (PMC8847458; doi:10.1038/s42003-022-03077-6)
Supplement: Supplementary file 3 — Description of Additional Supplementary Files [file 42003_2022_3077_MOESM3_ESM.pdf]

Description of Additional Supplementary Files

File name: Supplementary Video 1
Description: DeepLabCut kinematic model for healthy and spinal cord injured mice.

File name: Supplementary Video 2
Description: DeepLabCut ladder rung model for healthy and spinal cord injured mice.
