# Supplementary material for: A deep learning-based toolbox for Automated Limb Motion Analysis (ALMA) in murine models of neurological disorders
Source: Commun Biol. 2022 Feb 15;5:131. doi: 10.1038/s42003-022-03077-6 (PMC8847458; doi:10.1038/s42003-022-03077-6)
Supplement: Supplementary file 6 — Reporting Summary [file 42003_2022_3077_MOESM6_ESM.pdf]

nature portfolio

Corresponding author(s): F. Bareyre (florence.bareyre@med.uni-muenchen.de)

Last updated by author(s): 15.11.2021

Reporting Summary

Nature Portfolio wishes to improve the reproducibility of the work that we publish. This form provides structure for consistency and transparency in reporting. For further information on Nature Portfolio policies, see our Editorial Policies and the Editorial Policy Checklist.

Statistics

For all statistical analyses, confirm that the following items are present in the figure legend, table legend, main text, or Methods section.

n/a	Confirmed	
☐	☒	The exact sample size (*n*) for each experimental group/condition, given as a discrete number and unit of measurement
☐	☒	A statement on whether measurements were taken from distinct samples or whether the same sample was measured repeatedly
☐	☒	The statistical test(s) used AND whether they are one- or two-sided<br>*Only common tests should be described solely by name; describe more complex techniques in the Methods section.*
☒	☐	A description of all covariates tested
☒	☐	A description of any assumptions or corrections, such as tests of normality and adjustment for multiple comparisons
☐	☒	A full description of the statistical parameters including central tendency (e.g. means) or other basic estimates (e.g. regression coefficient) AND variation (e.g. standard deviation) or associated estimates of uncertainty (e.g. confidence intervals)
☐	☒	For null hypothesis testing, the test statistic (e.g. *F*, *t*, *r*) with confidence intervals, effect sizes, degrees of freedom and *P* value noted<br>*Give P values as exact values whenever suitable.*
☒	☐	For Bayesian analysis, information on the choice of priors and Markov chain Monte Carlo settings
☒	☐	For hierarchical and complex designs, identification of the appropriate level for tests and full reporting of outcomes
☒	☐	Estimates of effect sizes (e.g. Cohen's *d*, Pearson's *r*), indicating how they were calculated

Our web collection on statistics for biologists contains articles on many of the points above.

Software and code

Policy information about availability of computer code

Data collection	Data were collected using one GoPro 8 camera at 120 frames per second.
Data analysis	Data were analyzed using our ALMA toolbox as described in the paper for kinematic and ladder rung analysis.

For manuscripts utilizing custom algorithms or software that are central to the research but not yet described in published literature, software must be made available to editors and reviewers. We strongly encourage code deposition in a community repository (e.g. GitHub). See the Nature Portfolio guidelines for submitting code & software for further information.

Data

Policy information about availability of data

All manuscripts must include a data availability statement. This statement should provide the following information, where applicable:

- Accession codes, unique identifiers, or web links for publicly available datasets
- A description of any restrictions on data availability
- For clinical datasets or third party data, please ensure that the statement adheres to our policy

All data are available in the manuscript or in the supplementary materials. Raw data is availbale upon reasonable request to the corresponding author. All codes are provided for the use of ALMA

Field-specific reporting

Please select the one below that is the best fit for your research. If you are not sure, read the appropriate sections before making your selection.

- [x] Life sciences
- [ ] Behavioural & social sciences
- [ ] Ecological, evolutionary & environmental sciences

For a reference copy of the document with all sections, see nature.com/documents/nr-reporting-summary-flat.pdf

Life sciences study design

All studies must disclose on these points even when the disclosure is negative.

Sample size	No statistical methods were used to pre-determine sample size but our sample sizes are similar to those reported in previous publications. Bradley, P. M. et al. J. Exp. Med. 216, 2503–2514 (2019). Locatelli, G. et al. Nat. Neurosci. 21, 1196–1208 (2018). Jafari, M. et al. Nat. Neurosci. 24, 355–367 (2021).
Data exclusions	Pre-established exclusion criteria were used in this study. For SCI, mice were included only if they showed at least one point of improvement in the Basso Mouse Scale (BMS) over time. For TBI all mice were included. For EAE, mice were excluded if they did not develop disease.
Replication	All experiments in this study include at least 5 biological replicates. The number of replicates (ns) is mentioned in the text or figure legend.
Randomization	Mice were injured and followed over time for SCI and TBI. Mice were immunized for EAE and followed overtime. No randomization was necessary as there is no control or treatment groups. Only mice tested pre-injury and then injured and tested again at different time points post-injury or post-disease onset.
Blinding	Data collection and analysis was performed blind to the conditions of the experiment, unless this was not possible (for exemple, behavioral recording with the GoPro camera pre/post-injury/disease).

Reporting for specific materials, systems and methods

We require information from authors about some types of materials, experimental systems and methods used in many studies. Here, indicate whether each material, system or method listed is relevant to your study. If you are not sure if a list item applies to your research, read the appropriate section before selecting a response.

Materials & experimental systems

n/a	Involved in the study	
☒	☐	Antibodies
☒	☐	Eukaryotic cell lines
☒	☐	Palaeontology and archaeology
☐	☒	Animals and other organisms
☒	☐	Human research participants
☒	☐	Clinical data
☒	☐	Dual use research of concern

Methods

n/a	Involved in the study	
☒	☐	ChIP-seq
☒	☐	Flow cytometry
☒	☐	MRI-based neuroimaging

Animals and other organisms

Policy information about studies involving animals; ARRIVE guidelines recommended for reporting animal research

Laboratory animals	C57Bl6J mice, eight to sixteen weeks-old, were used for this study.
Wild animals	No wild animals were used in this paper.
Field-collected samples	No field-collected samples were used here.
Ethics oversight	All animal experiments were performed in accordance with the regulations of the local animal committee of the state of Bavaria (Regierung von Oberbayern).

Note that full information on the approval of the study protocol must also be provided in the manuscript.
