# Supplementary material for: A deep learning-based toolbox for Automated Limb Motion Analysis (ALMA) in murine models of neurological disorders
Source: Commun Biol. 2022 Feb 15;5:131. doi: 10.1038/s42003-022-03077-6 (PMC8847458; doi:10.1038/s42003-022-03077-6)
Supplement: Supplementary file 1 — Transparent Peer Review File [file 42003_2022_3077_MOESM1_ESM.pdf]

REVIEWERS' COMMENTS:

Reviewer #1 (Remarks to the Author):

I am satisfied that the authors have addressed all of my concerns. They have provided further analysis of some of the data and properties of their new tool. These additions greatly strengthened the tests for validity of this new tool.

Reviewer #2 (Remarks to the Author):

The authors have satisfactorily addressed my comments and concerns and I think that this manuscript will be a valuable addition to the field.
